# Overweight and obesity amongst Black women in Durban, KwaZulu-Natal: A ‘disease’ of perception in an area of high HIV prevalence

**DOI:** 10.4102/phcfm.v5i1.450

**Published:** 2013-02-19

**Authors:** Rynal Devanathan, Tonya M. Esterhuizen, Romona D. Govender

**Affiliations:** 1 Department of Family Medicine, University of KwaZulu-Natal, South Africa; 2 Programme of Biostatistics, Research Ethics and Medical Law, University of KwaZulu-Natal, South Africa

## Abstract

**Background:**

Overweight and obesity is an emerging health problem, particularly amongst urban Black women living in areas of high HIV prevalence. Understanding factors affecting this pandemic is essential to enable effective weight loss programmes to be implemented. This study explored urban Black women's perception of their body image against a backdrop of pre-existing non-communicable diseases (NCDs).

**Method:**

In this cross-sectional exploratory study 328 urban Black women were sampled systematically. Anthropometric measurements were conducted and women were interviewed using the Stunkard body image silhouettes as a tool to determine perception.

**Results:**

Most of the subjects (61%) were in the 40–59 years age group. Mean body mass index (BMI) was 37 (± 9.41 kg/m^2^) with over 90% being overweight or obese. Diabetes mellitus was the most common NCD, with a prevalence of 72%. Amongst the diabetic patients 7% were overweight and 64% obese. Perceived body image compared to derived BMI showed that women underestimated their body image across all weight categories. Over 40% indicated a normal to overweight preferred body image, with 99% of respondents associating the underweight silhouettes with disease and HIV infection.

**Conclusion:**

Urban Black women with underlying NCDs and living in an area of high HIV prevalence perceive themselves to be thinner than their actual BMI, which may be a barrier to weight loss management. This misperception may be used as a proxy risk marker for weight gain in urban Black women.

## Abstrait

**Surpoids et obésité parmi les femmes noires à Durban, KwaZulu-Natal: Une « maladie » de la perception dans une région à forte prévalence de VIH en Afrique du Sud**

**Contexte:**

Le surpoids et l'obésité sont un problème sanitaire émergeant, particulièrement chez les femmes noires vivant en milieu urbain dans des zones à forte prévalence de VIH. La compréhension des facteurs affectant cette pandémie est essentielle pour permettre la mise en place de programmes de perte de poids efficaces. Cette étude explore la perception qu'ont les femmes noires vivant en milieu urbain de leur image sur une toile de fond de maladies non transmissibles (MNT) préexistantes.

**Méthode:**

Pour cette étude transversale exploratoire, 328 femmes noires vivant en milieu urbain ont été sélectionnées de manière systématique. Des mesures anthropométriques ont été relevées et les femmes ont été interrogées en utilisant les figures de Stunkard en tant qu'instrument pour déterminer la perception.

**Résultats:**

La plupart des femmes (61%) étaient âgées de 40 à 59 ans. L'indice de masse corporelle (IMC) moyen était de 37 (± 9.41 kg/m^2^), plus de 90% de ces femmes étant en surpoids ou obèses. Le diabète non insulo-dépendant était la maladie non transmissible la plus courante avec une prévalence de 72%. Parmi les patientes diabétiques, 7% étaient en surpoids et 64% étaient obèses. L'image corporelle perçue comparée à l'IMC dérivée a montré que les femmes sous-estimaient leur image corporelle dans toutes les catégories de poids. Plus de 40% indiquaient préférer l'image d'un corps allant de normal à en surpoids, 99% des femmes interrogées associant les figures minces à la maladie et à l'infection du VIH.

**Conclusion:**

Les femmes noires vivant en milieu urbain avec des MNT sous-jacentes et vivant dans des zones à forte prévalence du VIH ont une perception de leur corps erronée, pensant qu'elles sont plus minces que leur IMC ne l'indique, ce qui pourrait être un obstacle à la perte de poids. Cette perception erronée peut être utilisée comme marqueur de risque de substitution pour une prise de poids chez les femmes noires vivant en milieu urbain.

## Introduction

The World Health Organisation (WHO) labels obesity as one of the most noticeable and yet most unheeded public health problems that threatens to devastate both developed and emerging countries.^1, 2^ Obesity has attained rampant proportions globally, with over one billion adults classified as overweight with a body mass index (BMI) ≥ 25 kg/m^2^, whilst at least 300 million are clinically obese with a BMI ≥ 30 kg/m^2^. Excessive weight has become a major contributor to the global burden of non-communicable diseases (NCDs) and debility, and is listed by the WHO as one of the 10 leading risk factors in developed countries which contribute to their national burden of disease. Overweight and obesity are modifiable risk factors, most of which can be reversed with adherence to weight loss programmes.^3^ Similar to other African countries, South Africa (SA) currently encounters an increasing burden of NCDs associated with overweight and obesity. Determinants of the obesity epidemic are complex, with women's perception of their body image gaining more attention in recent times.^4, 5^

Obesity and the associated NCDs have been considered problematic in affluent countries, where lifestyle and consumption of high-fat and carbohydrate-rich foods has resulted in obesity rates doubling for adults and tripling for children during 1980–2008 in the United States of America (USA).^6^ There is an increasing prevalence of obesity in nations that are undergoing socio-economic transition from being undeveloped to developed, for example China, Brazil and SA. They have an increased frequency of obesity across all economic levels and age groups, reported to be due to more disposable income and eating processed and unhealthy food.^7, 8^ SA is currently being challenged with a quadruple disease burden, which includes poverty-related diseases, NCDs, injuries and HIV infection and AIDS, with the estimated mortality profile for SA in 2000 reaching 21% of deaths due to communicable diseases, NCDs accounting for 37%, injuries 12%, and AIDS 30%, confirming that the socio-economic transition is being accompanied by NCDs.^9^

In the USA studies have shown that the relationship between body fat distribution and risk factors for disease differs between African Americans and Caucasians.^10^ Excessive weight and type 2 diabetes are closely related in both men and women across all ethnic groups.^11, 12^ The Nurse's Health Study demonstrated that risk of type 2 diabetes increased 40-fold when BMI increased from 22 kg/m^2^ to 35 kg/m^2^, contrary to the belief that ‘benign’ or ‘health’ obesity was without consequence.^11, 13^ In recognition of the negative consequences for many of its citizens, the Department of Health in SA has instituted a weight loss programme nationally, with training having been provided to nurses in clinics to advise people about the outcomes of excessive weight and how to lose it. This was implemented in 1990, but reports indicate that it is having little success in both rural and urban areas.^14^ This is evidenced by the 1998 South African Demographic and Health Survey (SADHS), which showed that the pattern of malnutrition in SA was one of over- rather than undernutrition.^15^ Overweight and obesity were estimated to be prevalent in a third of men and more than half of women respectively.^15^ Other significant contributory factors responsible for the obesity statistics seen in SA include accessibility to cheap, unhealthy foods, barriers to physical activity, and the beliefs and attitudes of Black women.^8^ In the study ‘Big is Beautiful’ the Black community health workers preferred a larger body size because of the attached positive connotations, which illustrates the impact of socio-cultural factors on body image.^16^

Ethnicity has a major impact on the incidence and pathogenesis of co-morbid disease in SA.^17^ The most vulnerable group is the Black female population, with an estimated prevalence of obesity between 31% and 34%.^15, 18^ In keeping with global trends, an association between obesity and hypertension, impaired glucose tolerance, diabetes mellitus, dyslipidaemia and ischaemic heart disease has been well documented in the White, Indian and Coloured (mixed ancestry) populations of SA. The situation in the Black ethnic group is complex, and has been the subject of much debate.^17^ In earlier studies it was shown that ischaemic heart disease and dyslipidaemia were less prevalent in the Black populace. This became foundational in establishing the concept of ‘benign’ or ‘healthy’ obesity, where it was assumed that obesity in this ethnic group was harmless and without consequence.^13^

The reasons for the emerging obesity epidemic are well documented. There is now a growing area of interest in the individual's perception of their body image, the subjective sense people have about their bodies, encompassing self-perception and attitudes towards their physical appearance. Perceptions are ideas that exist in the minds of people about how they are viewed by others.^16^ In the South African context, a women's perception of an ideal weight appears to be influenced by multiple factors including culture; amongst women classified as ‘Black’ it has been reported that being overweight and even obese increases their desirability by men and has many positive connotations.^16^ Puoane et al.,^15^ in an article on obesity research, reported that although the prevalence of obesity was the highest amongst Black woman in SA, fewer perceived themselves to be obese compared to their actual BMI. In the overweight and obese category, 21% self-reported being in this category whilst 57% had a BMI greater than 25 kg/m^2^.^15^

Studies on body perceptions may be contributing to the heightening awareness of the obesity epidemic in SA.^5^ Bays et al.^19^ have shown that misperception of body image may have preceded NCDs, which may be a barrier to weight loss management. In a recent study conducted in Khayelitsha, home to the largest urban black population in Cape Town, the prevalence of obesity was greater than 54% amongst Black women.^5^ The authors concluded that when people ‘should be avoiding risk factors for cardiovascular disease, especially obesity, many are becoming obese to avoid the stigma associated with being infected with HIV or having AIDS’.^5^

A perception that being overweight is desirable may be related to the AIDS epidemic, with a perception that thinness is associated with an HIV-positive status.^5^ A Western-based study indicates that African-American women who were infected with HIV attempted to gain weight to avoid being stigmatised.^20^ This preference for a big body size amongst Black women was also reported in an African-based study, where Puoane et al.^16^ described that being overweight meant that one was being taken good care of by one's husband. It also implied that the Black woman was ‘able to stir big pots and would not be blown away by the strong Cape winds’. On the other hand, being thin was equated with unhappiness, ill treatment and, most importantly, being infected with HIV or having AIDS.^16^ Matoti-Mvalo and Puoane^5^ showed that most Black urban women (69%) associated thinness with being infected with HIV or having AIDS, with 34% preferring to be overweight and 31% associating being overweight with good health.

South Africa is currently experiencing the largest HIV epidemic in the world, with an estimated 5.6 million South Africans being HIV positive in 2008.^21^ Of all the nine provinces, KwaZulu-Natal (KZN) has faced the greatest burden, with a prevalence of 16%.^21^ There is a dearth of research with regard to exploring perception of body image of urban Black women with pre-existing NCDs in an area of high HIV burden such as KZN.

The WHO and European Union emphasised the importance of looking at the social, cultural, political, physical and structural (environmental) influences for effective prevention and management of overweight and obesity.^1^, ^22^ It is important that the knowledge, attitudes and beliefs of people about their body image be investigated in planning intervention strategies to curb the obesity epidemic. This is a pioneer study which aimed to investigate the perception of Black women with underlying NCDs of their body image in an area of high HIV burden, thereby creating a platform for further research in developing culture-specific weight loss strategies in this group of women. The key objectives for this study were:

- to describe Black women's perception of their body image in relation to their actual BMI
- to describe the perceptions of Black women in relation to preferred body weight, healthy body weight, and one associated with HIV and AIDS
- to determine the prevalence of overweight and obesity amongst urban black women with the most commonly occurring NCD, type 2 diabetes.

### Significance of the work

Overweight and obesity are becoming a major public health concern due to the high burden of chronic diseases of lifestyle, particularly amongst urban Black women, against a backdrop of high HIV prevalence. Urban Black women may be reluctant to engage in a weight loss programme due to high levels of perception that thinness is associated with being infected with HIV or having AIDS. An appreciation of factors other than the biomedical may be needed to involve urban Black women in weight loss management. Clinicians attending to urban Black women with pre-existing NCDs would be upskilled in using a more socio-culturally specific approach to weight loss strategies.

## Method

### Design

A cross-sectional exploratory study was conducted at Wentworth Hospital, Durban, which is a district hospital with patients referred from primary healthcare facilities when more specialised treatment is needed. Black women between the ages of 18 and 70 years who attended the NCD clinic during the period June 2010 and October 2010 were selected for convenience. It was estimated that approximately 2000 Black women attended the NCD clinic in a year, this being obtained from the chronic patient booking register.

### Material and sample

A sample of approximately 10% (*n* = 328) will allow a 5% margin of error in estimating categorical population parameters with 95% confidence.^23^ A systematic sampling method was employed, which resulted in every sixth consenting woman participating in the study. If a woman did not consent then the next consenting women was selected, and the reasons for non-participation were recorded in the case record file, which was designed to capture information regarding demographic details, medical conditions, body weight, height, and body image pictures. Each record was completed by the principal investigator to ensure internal validity of the results.

Height was logged to the nearest 0.1 cm by means of a metal tape. The study participants were requested to use a thin hospital gown, and shoes and headgear were removed to ensure correct height estimations. The subject's weight was ascertained by means of a standardised automated load cell numerical scale with a ceiling weight of 136 kg (UC-Precision Health Scale, accurate to 0.05 kg). An equivalent scale was utilised to establish the masses of those weightier than 136 kg (Soehnle Medica, accurate to 500 g, maximum 150 kg). For subjects weighing above 150 kg, two equivalent scales were used, with a single foot on each one. The same scale was used throughout the study. The BMI, expressed as kg/m^2^, was calculated and recorded in the case file.

To determine the perceptions of the woman's own body image, the Stunkard body silhouettes were used. Figural stimuli were introduced by Stunkard et al. as an easy tool to administer self-reported measures of body image, and this has been validated and adapted for use in SA.^24, 25^ The reason for selecting these pictures as a method was to ensure that women who are illiterate were also able to express their perceptions of body image, thereby reducing selection bias.^24^ Administration of Stunkard's standard silhouettes required respondents to choose the silhouette that most closely resembled how they think they usually look, their preferred body image, a healthy body image, and one who has HIV or AIDS. The participants had to choose from body images 1 to 8, with the first silhouette representing thinness and the eighth one the largest body image.

### Data analysis

Analysis of the data was conducted by the Department of Biostatistics, University of KwaZulu- Natal. Standard descriptive statistics (numbers, proportions, and means) and standard deviations were used to describe the study cohort and their selection of body image silhouettes. The data were entered into a Microsoft Excel spreadsheet which was transferred onto the SPSS-16 statistical package for further analysis. The difference in proportions between the perceived body image and actual BMI was tested for significance using the Chi-squared test. A *p*-value < 0.05 was considered to be statistically significant at the 95% confidence interval.

## Ethical considerations

The Ethics Committee of the Faculty of Health Sciences at the University of KwaZulu-Natal approved the study. Permission was also obtained from the Chief Executive Officer of Wentworth Hospital and the KZN Provincial Department of Health. Participation in the study was voluntary, and issues of confidentiality were assured. The HIV status of the individuals was not requested as this was not part of the objective of this study.

## Results

The study sample comprised 328 Black women attending a NCD clinic, with ages ranging from 19 to 70 years and a mean of 49 years (s.d. ± 12.1). More than 60% of the women were between the ages of 40 and 59 years, representing the most productive age group of the population, with a minority (9%) in the 19–29 years range. The overall distribution of Black women's BMIs shows the prevalence of overweight to be 16% and of obesity 76% (Table 1).

**TABLE 1:** Distribution of Body Mass Index in women between the ages of 19 and 70 years.

BMI category	BMI value (kg/m^2^)	*N* = 328	%
Underweight	<18.50	1	0.3
Normal	18.50–24.99	26	7.9
Overweight	25.00–29.99	51	16
Obese	≥30.00	250	76

**Total**	**-**	**-**	**100**

*N*, Total number of participants.

Anthropometric measurements were obtained from all study subjects, with weights ranging between 40.4 kg to 193.9 kg. The mean weight was 88.4 kg (s.d. ± 23.44), and height in metres was 1.5 m (s.d. ± 0.1). The mean derived BMI was 37.2 (s.d. ± 9.4), which is equal to or greater than 30 kg/m^2^, a cut-off measure for obesity.

The women's perception of their body image in relation to their actual BMI shows that they underestimated their body image across all weight categories (Table 2): underweight 6.4% vs 0.3%, *p* = 0.0001; normal weight 14.33% vs 7.93%, *p* = 0.0092; overweight 52.13% vs 15.55%, *p* = 0.0001; and obese 27.13% vs 76.22%, *p* = 0.0001. Of those who were clinically obese (76%) according to their BMI, only 27% perceived themselves as having a large body image (silhouette numbers 7 and 8). Fifty two per cent of women perceived themselves to be overweight, but in fact only 15% had a BMI of 25 kg/m^2^ – 29.9 kg/m^2^.

**TABLE 2:** Subjects’ perception of body image in relation to their Body Mass Index.

Perceived body image	Underweight (≤18.5)		Normal (18.5–24.9)		Overweight (25–29.9)		Obese (≥30)		Total	

	*n*	%	*n*	%	*n*	%	*n*	%	*N*	%
Underweight	1	0.30	9	2.74	8	2.44	3	0.91	21	6.4
Normal	0	0	13	3.96	15	4.57	19	5.79	47	14.33
Overweight	0	0	4	1.22	26	7.93	141	42.99	171	52.13
Obese	0	0	0	0	2	0.61	87	26.52	89	27.13

**Total**	**1**	**0.30**	**26**	**7.93**	**51**	**15.55**	**250**	**76.22**	**328**	**100**

*N*, Total number of participants; *n*, Given as number of participants.

**FIGURE 1:**
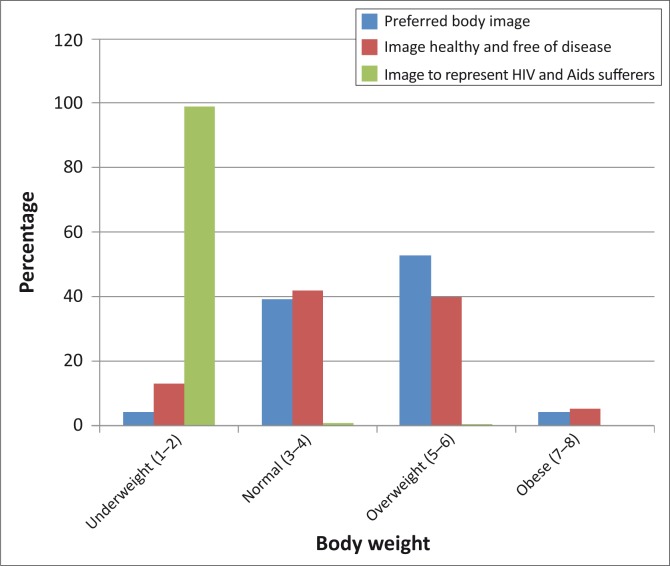
Subjects’ perception of body image in relation to preferred body image, healthy and free of disease body image, and body image representing HIV and AIDS sufferers.

Normal to overweight was indicated as a healthy body image free from disease (Figure 1), with approximately 99% of the women associating thinness with HIV and AIDS. The women with underlying NCDs preferred an overweight body image rather than a normal one.

All women who participated had one or more NCDs. Type 2 diabetes (72%) was the most prevalent medical condition encountered; in women who were obese 64% had diabetes mellitus compared to 7% in the overweight category. Hypertension alone or with co-morbidities was recorded in 34% of the population studied, with 15% having only osteoarthritis. A low percentage of chronic attendees (0.9%) had coronary heart disease as co-morbidity, and none reported having any form of cancer. The overweight and obese categories accounted for the largest percentage of the population with chronic diseases of lifestyle (Table 3).

**TABLE 3:** Medical conditions in relation to actual Body Mass Index.

Chronic medical conditions	Underweight		Normal		Overweight		Obese		Total	

	*n*	%	*n*	%	*n*	%	*n*	%	*N*	%
Hypertension	-	0	1	0.30	2	0.61	4	1.22	7	2.1
Hypertension And osteoarthritis	-	0	0	0		0	4	1.22	4	1.2
Diabetes mellitus	-	0	2	0.61	10	3.05	62	18.9	74	22.6
Diabetes mellitus and ischaemic heart disease	-	0	0	0	0	0	2	0.61	2	0.6
Diabetes mellitus and osteoarthritis	-	0	0	0	4	1.22	54	16.46	58	17.7
Hypertension and diabetes mellitus co-morbidity	-	0	1	0.30	9	2.74	53	16.16	63	19.2
Diabetes mellitus, hypertension and osteoarthritis	-	0	0	0	1	0.30	38	11.59	39	11.9
Ischaemic heart disease	-	0	0	0	0	0	1	0.30	1	0.3
Osteoarthritis	-	0	13	3.96	14	4.27	22	6.71	49	14.9
Other	1	0.30	9	2.74	11	3.35	10	3.05	31	9.5

**Total**	**1**	**0.3**	**26**	**7.9**	**51**	**15.6**	**250**	**76.2**	**328**	**100**

*N*, Total number of participants; *n*, Given as number of participants.

In those study participants with type 2 diabetes, 210 (64%) had an actual BMI of 30 kg/m^2^, with 136 (41%) misperceiving themselves to be thinner than their actual BMI (Table 3). Only 23% of women with type 2 diabetes perceived themselves to be obese, although 64% had a BMI ≥ 30 kg/m^2^ (Table 4). Those with type 2 diabetes who had a normal BMI were fewer than 1% (Table 3), compared to approximately 9% (Table 4) who perceived themselves to be of normal weight who had the same NCD profile.

**TABLE 4:** Medical conditions in relation to perceived body image.

Medical conditions	Underweight		Normal		Overweight		Obese		Total	

	*n*	%	*n*	%	*n*	%	*n*	%	*N*	%
Hypertension	2	0.61	1	0.30	4	1.22	0	0	7	2.1
Hypertension and osteoarthritis	0	0	0	0	2	0.61	2	0.61	4	1.2
Diabetes mellitus	2	0.61	10	3.05	45	13.72	17	5.18	74	22.6
Diabetes mellitus and ischaemic heart disease	0	0	1	0.30	1	0.30	0	0	2	0.6
Diabetes mellitus and osteoarthritis	0	0	6	1.83	29	8.84	23	7.01	58	17.7
Hypertension and diabetes mellitus co-morbidity	6	1.83	9	2.74	26	7.93	22	6.71	63	19.2
Diabetes mellitus, hypertension and osteoarthritis	0	0	3	0.91	24	7.31	12	3.66	39	11.9
Ischemic heart disease	1	0.30	0	0	0	0	0	0	1	0.3
Osteoarthritis	6	1.83	6	1.83	25	7.62	12	3.66	49	14.9
Other	5	1.52	11	3.35	14	4.27	1	0.30	31	9.5

**Total**	**22**	**6.7**	**47**	**14.3**	**170**	**51.8**	**89**	**27.1**	**328**	**100**

*N*, Total number of participants; *n*, Given as number of participants.

## Discussion

The Moscow Declaration of 2011, which stemmed from the First Global Ministerial Conference on Healthy Lifestyles and NCDs Conference in April 2011, stated that the rationale for action is due to growing worldwide problems associated with overweight and obesity.^26^ NCDs are important causes of premature deaths which affect the most susceptible and poorest populations, impacting on the lives of billions of people with financial implications that ruin individuals and families, particularly in low- and middle-income countries.^26, 27^ The neglected conditions of overweight and obesity are now being recognised as health challenges accompanying the socio-economic transition in SA, which places an additional and avoidable burden on the healthcare budget reserves due to the extent of the problem and its associated cosequences.^8^ SA's complex ethnic, social, economic and cultural influences have contributed to the quadruple burden of disease, with excess weight being a well-recognised risk factor for NCDs. Overweight and obesity have been contributing to this burden, highlighting the need to understand the factors fuelling this epidemic, such as environmental influences, socio-economic status, beliefs and attitudes, accessibility to cheap, unhealthy foods, and barriers to physical activity.^8, 27^ This study gives insight into understanding the perception of body image of Black women with NCDs and postulates possible reasons for reluctance for weight loss in an area of high HIV burden.

With a high prevalence of HIV infection in the area, these women demonstrated a large degree of misperception about their body image, with obese women showing the greatest variation in correct identification. This failure to correctly perceive their body image and to understand the possible resulting health consequences has led to a delay in the acceptance of government's weight loss strategy to curb NCDs. Of concern is that women with pre-existing NCDs, and specifically type 2 diabetes, perceived themselves smaller than their actual BMI, this being supported by the findings of Bays et al.^19^ These findings were also supported by Puoane et al.,^16^ who found that despite the prevalence of overweight and obesity being highest amongst Black women, few perceived themselves to be obese compared to their actual BMI. A higher frequency (64%) of obesity amongst those with type 2 diabetes was noted in this study, this being comparable to Rotchford and Rotchford's^28^ finding in Black women of 84%. This high prevalence of type 2 diabetes in the obese group is contrary to earlier studies which theorised that ‘benign or healthy obesity’ had no health consequences in the Black population.^13^

In the SADHS of 1998 it was reported that 27.1% of urban Black women were overweight and 33.8% were obese; these findings are lower than those of this study, which demonstrated a lower prevalence of overweight (16%) and a much higher one for obesity (76%).^15^ This difference could be due to the sample size and study population of both studies; however, a shift from overweight to obesity could be due to the degree of misperception of one's body size in the context of the Black culture, where being rounded has many positive connotations.^16^

It was shown across all weight categories that women perceived themselves thinner or smaller than their actual BMI. This is disconcerting given that these women belonged to a chronic clinic which practiced health promotion and healthy living. Although these women had prior knowledge of the health risks associated with being overweight and obese, they preferred to be overweight (53%) rather than of normal weight (39%). These percentages are higher than in the Matoti-Mvalo and Puoane^5^ study in Khayelitsha, Cape Town, where 33.5% preferred being overweight to normal weight (50%). This may be due to the lower prevalence of HIV in this district of Cape Town (6%) compared to KZN (16%).^21^ This behaviour could have major implications on healthcare costs in the future, owing to misperceptions of one's body image. In the ‘Big is Beautiful’ study the authors concluded that although the community health workers had prior knowledge of the disadvantages of being overweight and obese, they chose to be large and not to lose weight, compromising their ability to lead by example when instructing others.^16^

The women in this study correctly associated a normal body image with health and being free of disease, but nevertheless personally preferred a larger body image. The high level of stigmatisation of HIV infection in the area could account for the shift in preference towards a larger body image, therefore increasing the overweight and obesity statistics reported in the SADHS.^15^ In KZN, the epicentre of South Africa's AIDS pandemic, almost all of the women associated thinness with having HIV infection and AIDS, which could be due to the high HIV load in the region and most patients being well sensitised about this pandemic.^21^ This level of perception that thinness is associated with HIV infection and AIDS was higher than that reported by Matoti-Mvalo and Puoane (69%).^10^, ^16^

## Limitations of the study

The limitations include not obtaining the HIV status of the participants, which may have influenced the perception of their body image. The prevalence of overweight and obesity may have been overstated as the sample was drawn from an NCDs clinic, where many of the health problems are known to be associated with overweight. As this study was done in Durban and only Black women with NCDs were included, it may not be possible to extrapolate the results to all Black women and other ethnic groups in SA.

## Conclusion and recommendations

This study provides insight into the intricacies associated with weight loss strategies in vulnerable urban Black women with underlying NCDs who live in an area of high HIV prevalence. It shows the need to understand the complex issues associated with perception of body image amongst urban Black women, and highlights the challenges faced in engaging them in an effective weight loss management programme. This mixed perception amongst Black females could be perpetuating the current overweight and obesity crisis in KZN in particular, and in the rest of the country as well. SA could therefore anticipate a rise in the incidence of the metabolic syndrome and other NCDs in the future, due to the shift towards an obesogenic society.

The high level of misperception of body image occurred in a milieu of escalating HIV infection, which could be shifting Black women's preference for a more rounded body figure. Black women with NCDs will therefore be more reluctant to lose weight, despite knowing the health consequences of excess weight. Clinicians dealing with such individuals face a major hurdle unless a culture-specific approach is adopted to address the reasons for the weight status. Excess weight is a problem across all races and ages in SA, and unless there is a concerted effort on all platforms with all stakeholders to combat it, the associated health problems are going to increase the burden of disease faced by the resource-constrained Department of Health. In a country faced with a number of serious diseases (including AIDS and tuberculosis), every effort needs to be made to ensure that quality of life is optimised, premature deaths are minimised, and the citizens understand the consequences of their actions in living a healthy and long life.

Future studies should focus on addressing ways in which perceptions surrounding body image can be changed. Unlike in developed countries, women with HIV infection in SA are typically overweight to obese, and perceptions of body image in people living with HIV should be explored to determine their contribution towards an obesogenic society, as the misperception of body image may be enhancing the obesity epidemic.
